# Communications

**Published:** 1874-04

**Authors:** 


*Communications.*

The editors take great pleasure in acknowledging the reception of the following communications, which will appear at an early day:

Tetanus and Strychnia Poisoning, their Symptoms and Differential Diagnosis, by W. P. Wells, M. D., of Ohio; Is Diphtheria Contageous, by A. S. Payne, M. D., of Virginia; The Angina of the throat—Quinsey—Epidemic Tonsillitis a Distinct Disease, etc., etc., No. 1 and 2, by A. S. Payne, M. D., of Virginia; Sulphate of Atrophia in Ephidrosis Profusa, by C. H. Tidd, M. D., of Ohio; Hypodermic Medication, by J. C. Bishop, M. D., of Ohio; Strychnia Poisoning and its Diagnosis from Tetanus, by J. Q. A. Hudson, M. D. of Ohio; Coffiene in Headache, by T. Curtis Smith, M. D., of Ohio; Hydrate of Chloral, by A. S. Payne, M. D., of Virginia; On Gelseminum, by M. W. Morton, M.D., of Alabama; Broncho-Vesiculitis and Bronchial Rheumatism, by L. B. Anderson, M.D., of Virginia; Laryngo-Tracheotomy, by R. J. Preston, M.D., of Virginia; Imperforate Hymen, by T. M. Shaw, M.D., of Georgia; Report of a Case, by J. M. Lewis, M.D., of Mississippi;

Report of a Case, by Thos. S. Mitchell, M.D., of Georgia; The Rattle-Snake, its Poison and Antidote, by G. M. Rivers, M.D., of South Carolina; What was it? by Jno. W. Booth, M.D., of North Carolina; Fracture of the Clavical, by A. R. Kilpatrick, M.D., of Texas; and a package of Original Articles and Abstracts from S. M. Burnett, M.D., of Knoxville, Tenn.

In our next issue, owing to the large amount of original matter on hand, we will give more space to the original department. We trust our friends will continue to write. We will give each one a place and a cordial welcome in our pages. Write friends! We will give you room, even if it should be necessary to issue occasionally a few numbers filled entirely with original matter.

